# The effect of a bout of resistance exercise on skeletal muscle protein metabolism after severe fasting

**DOI:** 10.14814/phy2.14270

**Published:** 2019-11-05

**Authors:** Kohei Sase, Kohei Kido, Satoru Ato, Satoshi Fujita

**Affiliations:** ^1^ Faculty of Sport and Health Science Ritsumeikan University Kusatsu Shiga Japan

**Keywords:** Autophagy, fasting, resistance exercise

## Abstract

Resistance exercise (RE) activates the mechanistic target of rapamycin complex 1 (mTORC1) signaling pathway and increases muscle protein synthesis. Severe fasting induces 5′ adenosine monophosphate‐activated protein kinase (AMPK), which attenuates mTORC1 activation. However, the effect of RE on the response of mTORC1 signaling proteins after a period of severe fasting is unclear. We investigated the effect of RE on rat skeletal muscle protein metabolism after a period of severe fasting. We hypothesized that RE‐induced activation of mTORC1 signaling protein attenuates protein breakdown by autophagy. Male Sprague‐Dawley rats were divided into ordinary‐fed (C) and 72‐h fasting (F) groups. A bout of RE was replicated by percutaneous electrical stimulation in the right gastrocnemius muscle. The tuberous sclerosis complex 2 (TSC2) Ser1387 and autophagy marker of microtubule‐associated protein 1A/1B‐light chain 3‐II (LC3B‐II) expression of the F group increased twice that of the C group in sedentary state (*P* < 0.05). RE activated the mTORC1 signaling pathway in both groups (*P* < 0.05); however, in the F group, the magnitude of p70S6K (Thr389) phosphorylation was lower by 40% of that of the C group (*P* < 0.05). Protein synthesis after RE was increased by 50% from the level at sedentary state in the C group (*P* < 0.05), but not in the F. In the F group, the expression of LC3B‐II at 3 h after RE was decreased by almost 25% from the level at sedentary state (*P* < 0.05). Our results suggest that RE suppressed fasting‐induced autophagy but did not increase protein synthesis during severe fasting in rat skeletal muscle.

## Introduction

Malnutrition stimulates muscle atrophy, and it is an important problem in certain conditions. For example, the food intake of older persons in their seventies decreases by nearly 30% compared to the intake of younger persons in their twenties (Morley 1997). In patients who undergo gastrectomy, postoperative food intake decreases and their skeletal muscle mass is decreased by 6.2% compared with that in the preoperative period (*P* < 0.001) (Yamaoka et al. 2015). Weight‐classed athletes lose body mass, which decreases by 13.2% compared with that mass before weight loss by severe food restriction (Kukidome et al. 2008). The reduction in skeletal muscle mass leads to reduction in muscle strength, quality of life, postoperative recovery, and competitive performance (Saeki et al. 2001; Berkovich et al. 2019). Muscle atrophy due to malnutrition is an important problem to understand, but the mechanism that prevents muscle loss during the period of malnutrition remains unclear.

Skeletal muscle mass is regulated by the balance between muscle protein synthesis and breakdown. Although food intake and exercise have been shown to stimulate protein synthesis, even a brief period of malnutrition promotes protein breakdown and reduces protein synthesis (Norton and Layman 2006). Thus, reducing the changes in protein breakdown and synthesis during fasting is critical to prevent muscle atrophy during states of malnutrition.

In mammals, skeletal muscle is the largest protein reservoir. Malnutrition activates skeletal muscle macroautophagy, hereafter referred to as autophagy, to supply amino acids, especially alanine, to be used for gluconeogenesis (Parrilla 1978b; Takagi et al. 2016). Autophagy breaks down proteins to create double‐membrane structures (phagophores) that envelop the cytoplasm and organelles (autophagosomes) for delivery to the lysosomes, where they are broken down further (Kabeya et al. 2000). During energy deprivation, the energy substrate is decreased, and it activates 5′‐adenosine monophosphate‐activated protein kinase (AMPK), phosphorylates unc‐51–like kinase 1 (ULK1) at Ser317, and induces a generation of phagophores (Mizushima et al. 2004; Kim et al. 2011). An in vivo study indicate that a 72‐h fast increases the expression level of microtubule‐associated protein 1A/1B‐light chain 3‐II (LC3b‐II) (a marker of autophagosomes) 10‐100 times, compared with fed state (*P* < 0.05) (Ogata et al. 2010). Meanwhile, the mechanistic target of rapamycin complex 1 (mTORC1) phosphorylates ULK1 at Ser757 and inhibits the generation of phagophores (Kim et al. 2011; Jung et al. 2011). Based on the above, we assumed that the activation of mTORC1 is effective for reducing fasting‐induced muscle atrophy.

Resistance exercise (RE) induces protein synthesis through the mTORC1 signaling pathway (Ato et al. 2019). mTORC1 phosphorylates ribosomal protein S6 kinase beta‐1 (p70S6K) at Thr389, and p70S6K activates S6 ribosomal protein (rpS6) to initiate translation (Ma and Blenis 2009). In an animal study, Takamura et al. (2016) reported that RE replicated by percutaneous electrical stimulation to the gastrocnemius muscle activates mTORC1 signaling proteins and skeletal muscle protein synthesis increased 1.5 times that of the sedentary state (Takamura et al. 2016). In another study, a bout of RE reduced the LC3b‐II expression by 50% of that of the sedentary state (*P* < 0.05) in human skeletal muscle (Fry et al. 2013). However, the effect of RE on skeletal muscle autophagy and mTORC1 signaling proteins after a period of fasting has not been reported. Therefore, the purpose of this study was to investigate the effect of a bout of RE on rat skeletal muscle autophagy and mTORC1 signaling proteins after 72 h of fasting. We hypothesized that RE‐induced activation of mTORC1 signaling protein attenuates protein breakdown by autophagy.

## Materials and Methods

### Animals and experimental protocol

The study protocol was approved by the Ethics Committee for Animal Experiments at Ritsumeikan University. Thirty 11‐week‐old Sprague‐Dawley rats were obtained (330–360 g; Japan SLC, Inc., Kyoto, Japan). The rats were housed for 1 week in a temperature‐controlled space (22–24°C) with a 12‐h light and 12‐h dark cycle and received food and water ad libitum until the study started. After 1 week of acclimation, animals were divided into two groups: ordinary‐fed rats continued to receive food and water ad libitum (C group; n = 18) and rats that underwent 72 h of fasting (F group; *n* = 18). The C and F groups were exercised after 12 and 72 h of fasting, respectively. Before RE (sedentary state), at 0 after RE, and 3 h after RE, the rats were sacrificed, and fat, blood, and gastrocnemius muscle samples were obtained.

### Resistance exercise mimics muscle contraction model

Under isoflurane anesthesia, the hair of the right lower leg of each rat was shaved off and cleaned with an alcohol wipe. Rats were then positioned with their right foot on a footplate in prone posture and the ankle joint at 90°. The gastrocnemius muscle was stimulated percutaneously with electrodes (Vitrode V, Ag/AgcCl; Nihon Kohden, Tokyo, Japan). Electrodes were cut into 10 × 5–mm sections and connected to an electric stimulator and isolator (SS‐104J; Nihon Kohden). The right gastrocnemius muscle was isometrically exercised. The exercise session was conducted with ten 3‐sec contractions with 7‐sec intervals between contractions for 5 sets and 3‐min rest intervals between sets. The voltage (~30 V) and stimulation frequency (100 Hz) were adjusted to produce maximal isometric tension. The left gastrocnemius muscles were not used for analysis.

### Determination of plasma‐free amino acids

Blood samples were drawn into vacuum tubes containing disodium ethylenediaminetetraacetate dihydrate, and after gently tilting the samples to allow mixing, they were immediately cooled on ice. Samples were centrifuged at 8600 *g* for 15 min. At 4 min, the supernatant plasma was collected. All samples were then stored at −80°C.

Supernatant plasmas were re‐centrifuged at 7000 *g* for 10 min at 4°C. The supernatant was mixed with 15% sulfosalicylic acid and re‐centrifuged at 14,000 *g* for 60 min at 4°C using an ultrafiltration filter. After re‐centrifugation, the lower layers were collected and taken as samples after protein removal. Amino acid concentrations were analyzed using a high‐speed analyzer (L‐8900; Hitachi, Tokyo, Japan). Amino acids were separated using ion exchange chromatography and were detected spectrophotometrically after post‐column reaction with ninhydrin. Forty types of amino acids and related molecules were measured.

### Western blotting analysis

Muscle samples were homogenized in a homogenization buffer‐containing radioimmunoprecipitation assay (RIPA) (Cell Signaling Technology, Danvers, MA, USA), phosphatase inhibitor cocktail (Roche, Germany), and protease inhibitor cocktail (Sigma‐Aldrich, St. Louis, MO, USA). Homogenates were centrifuged at 10,000 *g* for 10 min at 4°C. The supernatant was removed, and the protein concentration was measured using a protein assay kit (Wako, Osaka, Japan). All samples were diluted in 3 × sample buffer (1.0% vol/vol beta mercaptoethanol; 4.0% wt/vol sodium dodecyl sulfate (SDS); 0.16 mol/L Tris‐HCl, pH 6.8; 43% vol/vol glycerol; 0.2% wt/vol bromophenol blue) and boiled at 95°C for 10 min. Using 8–15% SDS‐polyacrylamide gels, 20 *µ*g of protein was separated by electrophoresis, transferred to polyvinylidene difluoride by electrophoresis, and subsequently transferred to polyvinylidene difluoride membranes. After the transfer, the membranes were washed in Tris‐buffered saline containing 0.1% polyoxyethylene sorbitan monolaurate (Tween 20; Wako, Osaka, Japan) (TBST), and membranes were then blocked with 5% skim milk in TBST for 1 h at room temperature. After blocking, the membranes were washed in TBST and incubated overnight at 4°C, with the primary antibodies diluted 1:1000 with 5% bovine serum albumin in TBST.

In this study, primary antibodies used were phosphorylation p70S6K (Thr389; Cell Signaling Technology), total p70S6K (Cell Signaling Technology), phosphorylation S6 ribosomal protein (Ser240/244; Cell Signaling Technology), total S6 ribosomal protein (Cell Signaling Technology), LC3B (Cell Signaling Technology), p62 (SQSTM1; MBL, Japan), total ULK1 (Cell Signaling Technology), phosphorylation ULK1 (Ser317; Cell Signaling Technology), phosphorylation ULK1 (Ser757; Cell Signaling Technology), phosphorylation AMPK (Thr172; Cell Signaling Technology), phosphorylation Tuberin/TSC2 (Thr1462; Cell Signaling Technology), phosphorylation Tuberin/TSC2 (Ser1387; Cell Signaling Technology), and total AMPK (Cell Signaling Technology). The membranes were then washed again in TBST and incubated for 1 h at room temperature with the appropriate secondary antibodies diluted 1:3000 with 1% skim milk in TBST.

The membranes were then processed using enhanced chemiluminescence (RPN2106; GE Healthcare, Buckinghamshire, UK). Enhanced chemiluminescence signals on the immunoblots were detected and measured using a Fuji LAS3000 luminescent imaging system (Las‐3000; Fujifilm, Tokyo, Japan) with MultiGauge software version 3.0 (Fujifilm). Band intensities were quantified using ImageJ 1.50f (National Institute of Health, Bethesda, MD, USA).

### Protein synthesis rate

Muscle protein synthesis was measured using the in vivo surface sensing of translation (SUnSET) method, as described previously (Goodman et al. 2011; Kido et al. 2016). For this, 0.04 mmol puromycin/g body weight (Calbiochem, Merck Millipore, Billerica, MA, USA) diluted with a 0.02‐mol/L phosphate‐buffered saline stock solution was injected into the rats intraperitoneally after 5 min of anesthesia, and the muscle was removed exactly 15 min after puromycin administration. Following homogenization and centrifugation at 3800 *g* for 3 min at 4°C, the supernatant was collected and processed for western blotting. A mouse monoclonal antipuromycin antibody (Merck Millipore, Billerica, MA, USA) was used to detect puromycin incorporation, which was determined as the sum of the intensities of all protein bands in the western blot.

### Statistical analysis

Sample sizes were determined by a power analysis based on a previous study in our laboratory (Takamura et al. 2016), (Kido et al. 2016). The sample size of *n* = 6 per group was enough to detect a 20% change in protein synthesis rate and significant change in p70S6K and rpS6 phosphorylation at 3 h after RE (*n* = 4‐6 is needed to provide 95% power). All data are presented as mean ± standard error. Significant differences among morphological changes, plasma glucose, and amino acids were determined by t‐test. Within signaling proteins, two‐way analysis of variance (ANOVA) was used to evaluate change. When ANOVA revealed a significant interaction or main effects (Fast × Time), the Bonferroni correction was performed as a *post hoc* analysis to identify the differences. The differences among protein synthesis rate were evaluated by one‐way ANOVA and Bonferroni correction was performed as a post hoc analysis to identify the differences (JMP version 10.0.0; SAS, Cary, NC, USA). All differences were determined to be significant at *P* < 0.05.

## Results

### Morphological changes

Body weight, epididymis fat weight (absolute), and gastrocnemius muscle wet weight decreased in the F group, compared with those in the C group (*P* < 0.05) (Table 1).

**Table 1:** Morphological changes in experimental animals.

Group	Body weight (g)		Epididymis fat		Gastrocnemius muscle	
	Pre	Post	Absolute weight (mg)	Epididymis fat per body weight (mg/g)	Absolute weight (mg)	Gastrocnemius muscle per body weight (mg/g)
C	344.9 ± 2.8	346.3 ± 2.5	3353.8 ± 200.7	10.3 ± 0.6	1734.9 ± 28.8	5.3 ± 0.1
F	345.4 ± 1.9	297.8 ± 1.9*	2933.1 ± 244.9*	9.8 ± 0.8	1636.9 ± 22.6*	4.4 ± 0.1*

Values are mean ± standard error.

* *P* < 0.05, vs. C group.

### Effect of fasting on plasma glucose and amino acids

Total amino acids were the sum of Tau, Thr, Ser, Asp, Gly, Ala, Cit, Val, Cys, Met, Ile, Leu, Tyr, Phe, Trp, Orn, Lys, His, Arg, and Pro. Glucogenic amino acids were the sum of Ser, Asp, Thr, Gly, Ala, Val, Cys, Met, Ile, Tyr, Trp, His, and Arg. Gluconeogenic amino acids did not decrease after 72 h of fasting; however, plasma glucose and plasma total amino acids decreased in the F group, compared with those in the C group (*P* < 0.05) (Table 2).

**Table 2:** Effect of fasting on amino acids and glucose.

Group	Blood glucose (mg/dL)	Plasma amino acids (*µ*mol/L)	Plasma glucogenic amino acids (*µ*mol/L)
C	111.8 ± 5.7	3457.2 ± 115.0	1885.3 ± 92.4
F	81.1 ± 6.0*	3080.3 ± 80.8*	1852.5 ± 42.9

Values are mean ± standard error.

* *P* < 0.05, vs. C group.

### Autophagic marker LC3B‐II and adaptor protein p62

**Figure 1:**
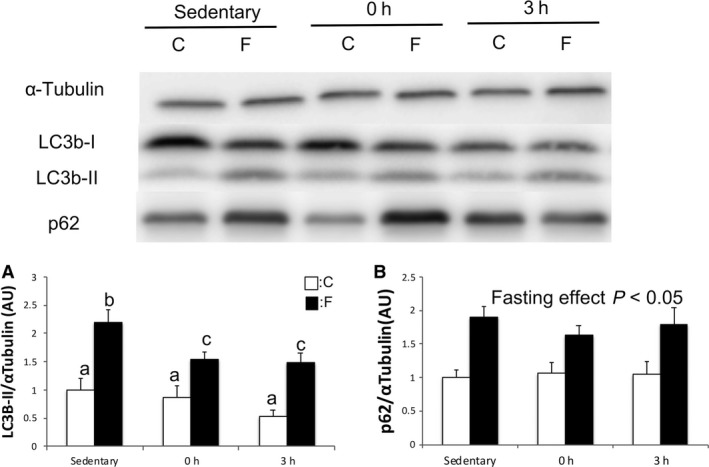
Changes in the expression of autophagy‐related proteins in rat gastrocnemius muscle. (A) LC3‐II and (B) p62 at sedentary state, 0 h after RE, and 3 h after RE. Values are mean ± standard error. *P* < 0.05, among different characters. All bars were analyzed by two‐way analysis of variance followed by Bonferroni post hoc analysis for multiple comparisons. LC3B‐II, light‐chain 3‐II; RE, resistance exercise.

The expressions of the autophagic marker of LC3b‐II and p62 are shown in Figure 1. Expressions of LC3B‐II and p62 were significantly increased by 72 h of fasting. At 0 and 3 h after RE, LC3b‐II expression was significantly decreased compared with the sedentary state in the F group (*P* < 0.05). However, p62 had no change after RE.

### mTORC1 pathway

**Figure 2:**
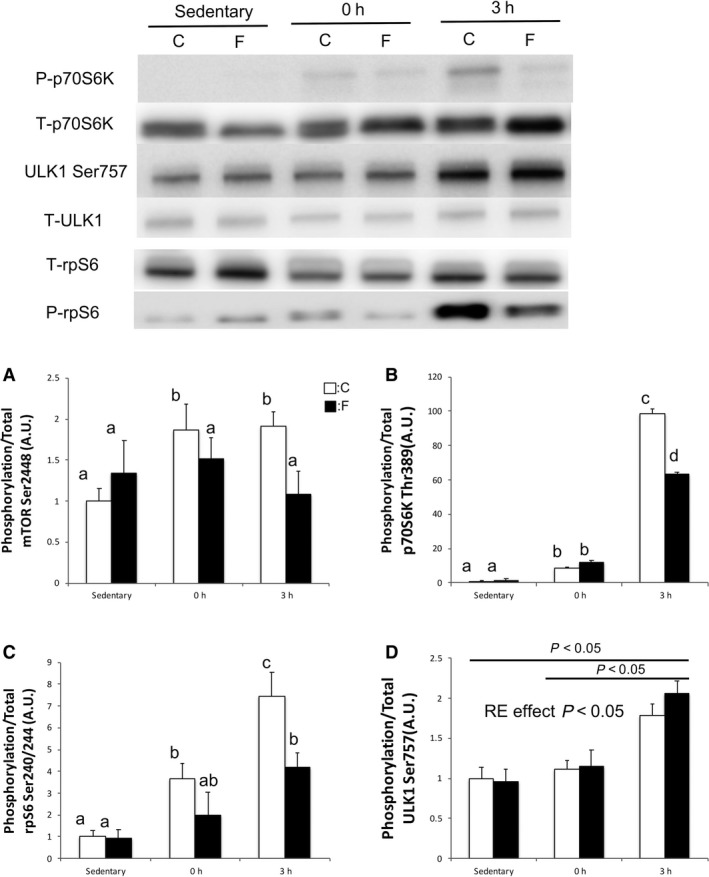
Changes in the phosphorylation of mTOR, p70S6K, rpS6, and ULK1 in the gastrocnemius muscle. Protein expression of phosphorylated mTOR‐Ser2448 (A), p70S6K‐Thr389 (B), rps6‐Ser240/244 (C), and ULK1‐Ser757 (D) were measured by western blotting after sedentary state, 1 h after RE, and 3 h after RE. Values are means ± standard error. *P* < 0.05, among different characters. All bars were analyzed using two‐way analysis of variance followed by Bonferroni post hoc analysis for multiple comparisons. mTOR, mechanistic target of rapamycin complex; p70S6K, phosphorylates ribosomal protein S6 kinase beta‐1; rpS6, S6 ribosomal protein; ULK1, unc‐51‐like kinase 1; RE, resistance exercise.

In the sedentary state, phosphorylation of mTOR‐Ser2448, p70S6K‐Thr389, ULK1‐Ser757, and rpS6‐Ser240/244 showed no significant difference between the F and C groups (Fig. 2A–D). The phosphorylation of p70S6K‐Thr389 was increased at 0 and 3 h after RE, compared with that during the sedentary state in both groups (*P* < 0.05). However, in the F group, the phosphorylation of p70S6K‐Thr389 and S6rp‐Ser246/244 decreased compared with that in the C group (*P* < 0.05) (Fig. 2B and C). The phosphorylation of ULK‐1‐Ser757 increased at 3 h after RE in both groups (*P* < 0.05) (Fig. 2D).

### Upstream of autophagy

**Figure 3:**
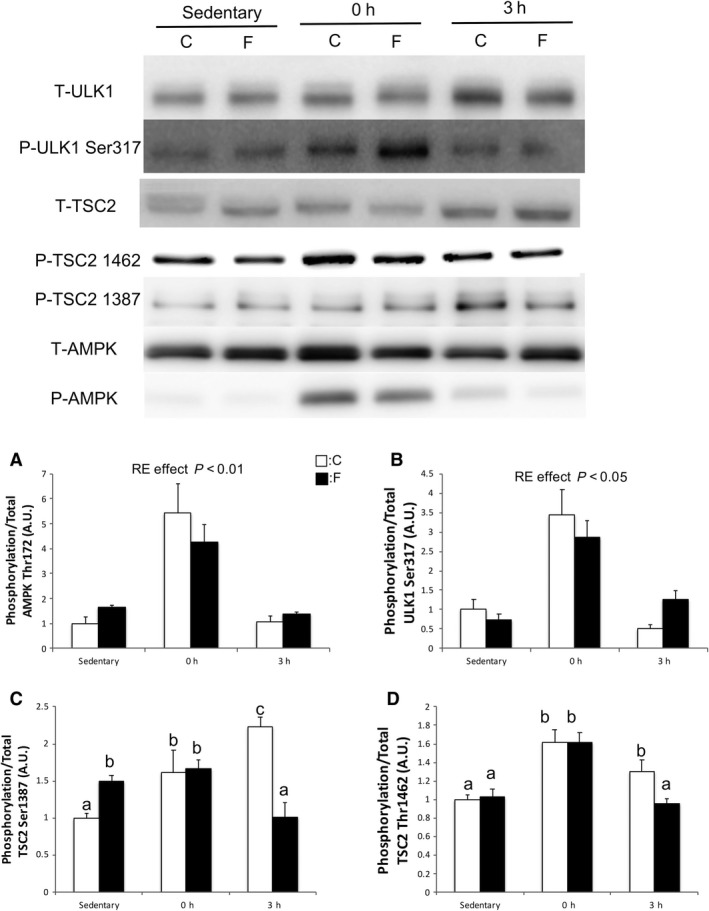
Changes in the phosphorylation of AMPK, ULK1, and TSC2 in the gastrocnemius muscle. Protein expression of phosphorylated AMPK‐Thr172 (A), ULK1‐Ser317 (B), TSC2‐Ser1387 (C), and TSC2‐Thr1462 (D) were measured by western blotting after sedentary state, 1 h after RE, and 3 h after RE. Values are means ± standard error. *P* < 0.05, among different characters. All bars were analyzed by two‐way analysis of variance followed by Bonferroni post hoc analysis for multiple comparisons. AMPK, 5′‐adenosine monophosphate–activated protein kinase; ULK1, unc‐51‐like kinase 1; TSC2, tuberous sclerosis complex 2; RE, resistance exercise.

The phosphorylation of AMPK‐Thr172 and ULK1‐Ser317 increased 0 h after RE, compared with that during the sedentary state in both groups (*P* < 0.05) (Fig. 3A and B). The phosphorylation of TSC2‐Ser1387 was significantly increased in the sedentary state compared with that in the C group. However, phosphorylation of TSC2‐Ser1387 was significantly decreased in the F group 3 h after RE (Fig. 3C).

### Protein synthesis rate

**Figure 4:**
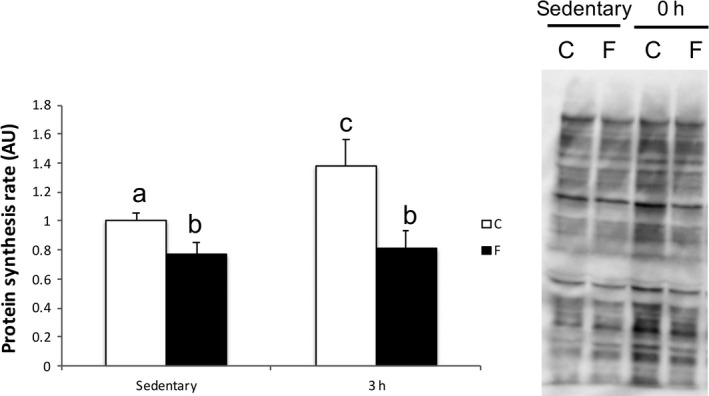
Muscle protein synthesis at sedentary state and 3 h after resistance exercise. Values are mean ± standard error. *P* < 0.05 among different characters. All bars were analyzed by two‐way analysis of variance followed by Bonferroni post hoc analysis for multiple comparisons.

Protein synthesis rate by puromycin decreased after 72 h of fasting in the sedentary state and at 3 h after RE (*P* < 0.05) (Fig. 4). In the C group, protein synthesis increased 3 h after RE compared with that during the sedentary state (*P* < 0.05); however, the F group was not changed by RE.

## Discussion

In this study, we investigated the effect of a bout of RE on rat skeletal muscle protein metabolism after 72 h fasting. Results show that fasting increased skeletal muscle autophagy and induced muscle atrophy, concomitant with decreased plasma glucose, total amino acid, and alanine concentration as well as protein synthesis at rest. On the contrary, RE significantly suppressed fasting‐induced autophagy. The activation of mTORC1 signaling pathway was increased by RE in both states; however, the magnitude was significantly decreased in the F group compared with that in the C group. Furthermore, RE‐induced protein synthesis did not increase after 72 h of fasting compared with that in the fed state.

In rats, 24 h fasting has been reported to deplete body glycogen and gluconeogenesis increased in proportion to fasting time (Parrilla 1978a). However, in this study, glucogenic amino acids were not changed by fasting. Previous studies reported that alanine is mainly used for gluconeogenesis, and subsequently, plasma alanine is decreased by fasting (Parrilla 1978a; Sato et al. 2016). In the current study, 72 h of fasting decreased plasma glucose and alanine. Hence, it seems that skeletal muscle breakdown occurred to maintain glucose homeostasis.

Several studies reported that fasting activates autophagy in the skeletal muscle of mammals (Mammucari et al. 2008; Ogata et al. 2010). In the present study, LC3b‐II and p62 expressions were used as a marker of autophagy. LC3b‐II exists as LC3b‐I in non‐stimulated condition. When autophagy is activated, LC3b‐I adheres to autophagosomes and becomes LC3b‐II after lipid modification (Kabeya et al. 2000). p62 can bind to LC3b and is degraded by autophagy; therefore, p62 is known as a marker of autophagic flux (Wang et al. 2006). In previous studies, the expression of p62 was increased two‐fold by severe fasting; however, the expression of p62 changed not only by activation of autophagy (Kuusisto et al. 2001; Nakaso et al. 2004; Ogata et al. 2010). The accumulation of p62 indicates the accumulation of phagophore and phagophore that is not yet degraded by lysosome. In the present study, in the F group, the sedentary state of LC3b‐II and p62 expression were increased two‐fold, compared with those in the C group (*P* < 0.05). These results support previous findings and show that prolonged fasting activated autophagy (Ogata et al. 2010).

According to in vivo studies, AMPK–Thr172 is activated by starvation, and AMPK phosphorylates ULK1–Ser317, consequently induces activation of autophagy when the body is experiencing starvation (Belkhou et al. 1991). In this study, 72 h of fasting induced autophagy. However, AMPK‐Thr172 and ULK1‐Ser317 were not changed by fasting. Similar to the findings of the in vivo study, there is no collective view of the phosphorylation of AMPK‐Thr172 and induction of autophagy by the AMPK–ULK1 signaling pathway (Gonzalez et al. 2004; Jamart et al. 2013; Schwalm et al. 2015). As a result, we need further studies to clarify the relationship between AMPK and induction of autophagy in vivo.

Previous studies have demonstrated that using electrical stimulation to replicate RE accurately stimulates mTORC1 signaling and skeletal muscle protein synthesis, and long‐term training can induce muscle hypertrophy (Kido et al. 2016; Ogasawara et al. 2016). In this study, a bout of RE significantly increased the phosphorylation of downstream targets of mTORC1 compared with the sedentary state in both groups, as reported in previous studies (Kido et al. 2016; Ogasawara et al. 2016). However, the magnitude of phosphorylation of p70S6K and rpS6 in 3 h after RE was decreased by 40% in the F group compared with that in the C group. This suppression seemed to occur through the phosphorylation of TSC2‐Ser1387. A recent study reported that the phosphorylation of TSC2‐Ser1387 suppresses mTORC1 activation (Thomson et al. 2008; Xu et al. 2012). In this study, the phosphorylation of TSC2‐Ser1387 was increased 1.5 times by fasting in the sedentary state (*P* < 0.05). Therefore, RE‐induced mTORC1 activation may be attenuated by TSC2 activation.

A bout of RE increased muscle protein synthesis in the C group, whereas no change in muscle protein synthesis was observed in the F group after RE. Therefore, RE after severe fasting interferes with acute RE‐induced muscle protein anabolism. A recent study proposed that insulin‐induced protein synthesis response is suppressed during low amino acid availability, and protein synthesis depends on amino acid availability (Bell et al. 2005). In this study, prolonged fasting decreased plasma amino acid concentration; hence, decreased amino acid availability may have suppressed the muscle protein synthesis after a bout of RE.

In the F group, the expression of LC3b‐II was 30% decreased 3 h after RE compared with the sedentary state (*P* < 0.05), which suggests that RE suppressed fasting‐induced formation of phagophore and that phagophore created before RE was degraded. However, the expression of p62 was not changed by RE. The main process of activation of autophagy is the formation of autophagosome; therefore, we have concluded that RE suppresses fasting‐induced autophagy. A study reported that mTORC1 activation phosphorylates ULK1–Ser757 and subsequently suppresses the formation of autophagosome (Kim et al. 2011). In this study, the phosphorylation of ULK1–Ser757 was increased two‐fold (P < 0.05), and this response mirrored p70S6K–Thr389 phosphorylation. Therefore, RE‐induced mTORC1 activation may have suppressed fasting‐induced autophagy.

### Limitations

RE, which was used in this study, does not mimic the systemic physiological changes; however, a study reported that acute electrical stimulation increases protein synthesis and chronic electrical stimulation induces muscle hypertrophy (Ogasawara et al. 2016). In a previous study, we focused on the effect of muscle contraction‐induced mTORC1 activation during severe fasting. Hence, the use of this model in this study was reasonable. The purpose of this study was to investigate the effect of a bout of RE on autophagy. To obtain measurable results, the length of fasting was extreme and cannot be sustained for longer periods.

## Conclusion

Our results suggest that RE suppressed fasting‐induced autophagy but did not increase protein synthesis in rat skeletal muscle during severe fasting. Based on the results of the present study, we can conclude that RE attenuates muscle protein breakdown by autophagy; therefore, chronic RE may attenuate muscle atrophy during food restriction state that induces muscle atrophy. Therefore, in future studies, we need to evaluate the effect of chronic RE on skeletal muscle anabolism and hypertrophic response during food restriction state that causes muscle atrophy.
